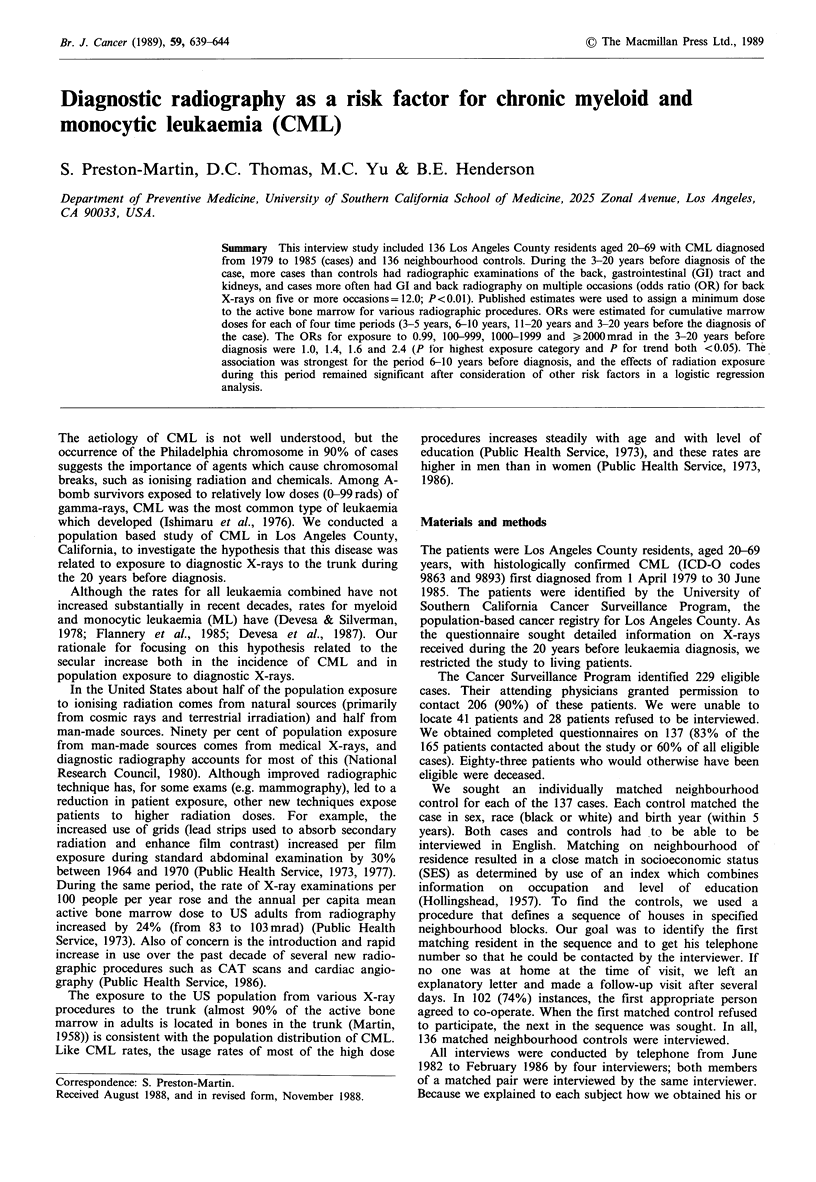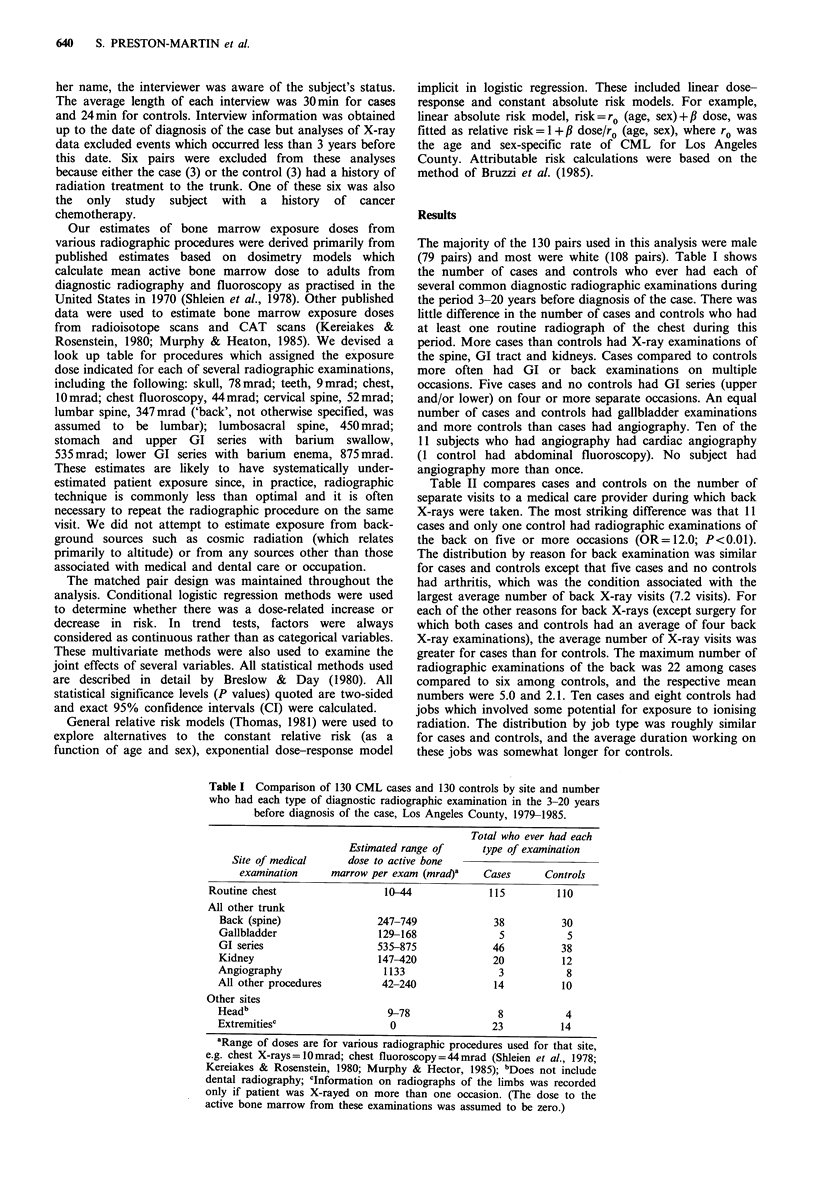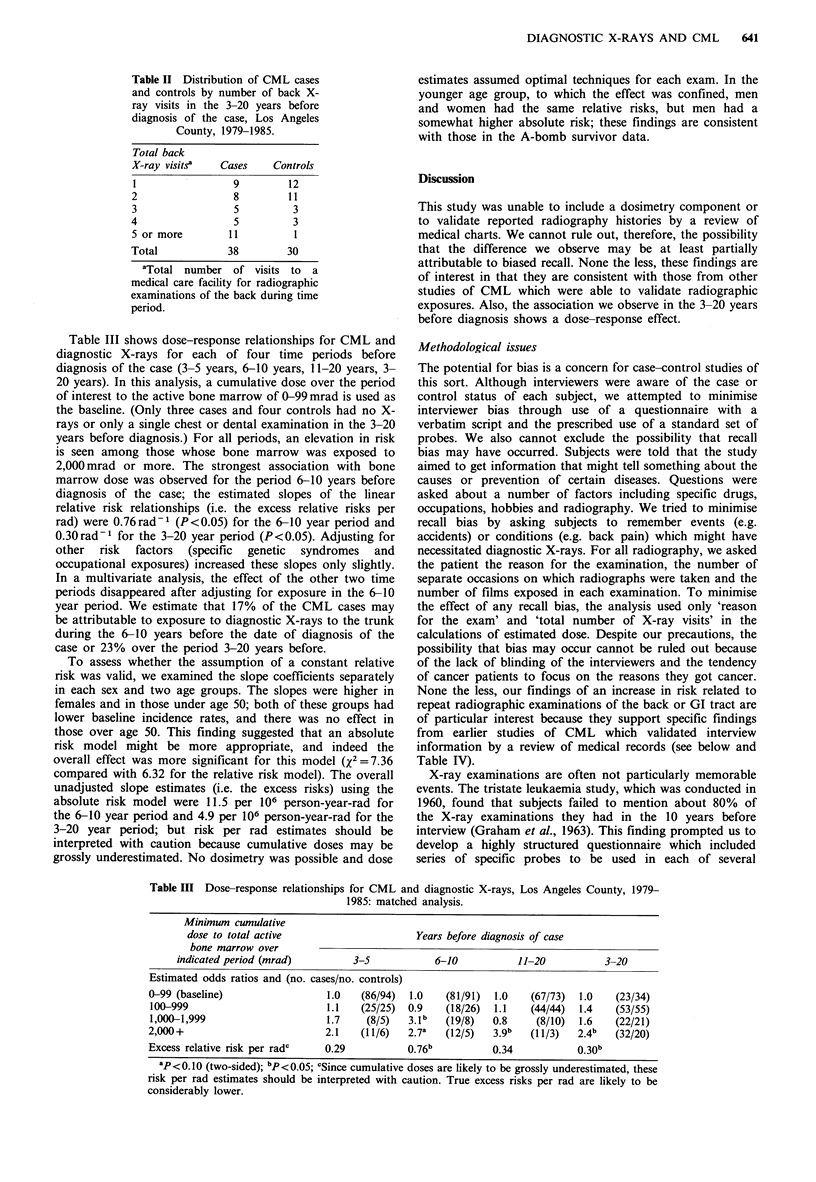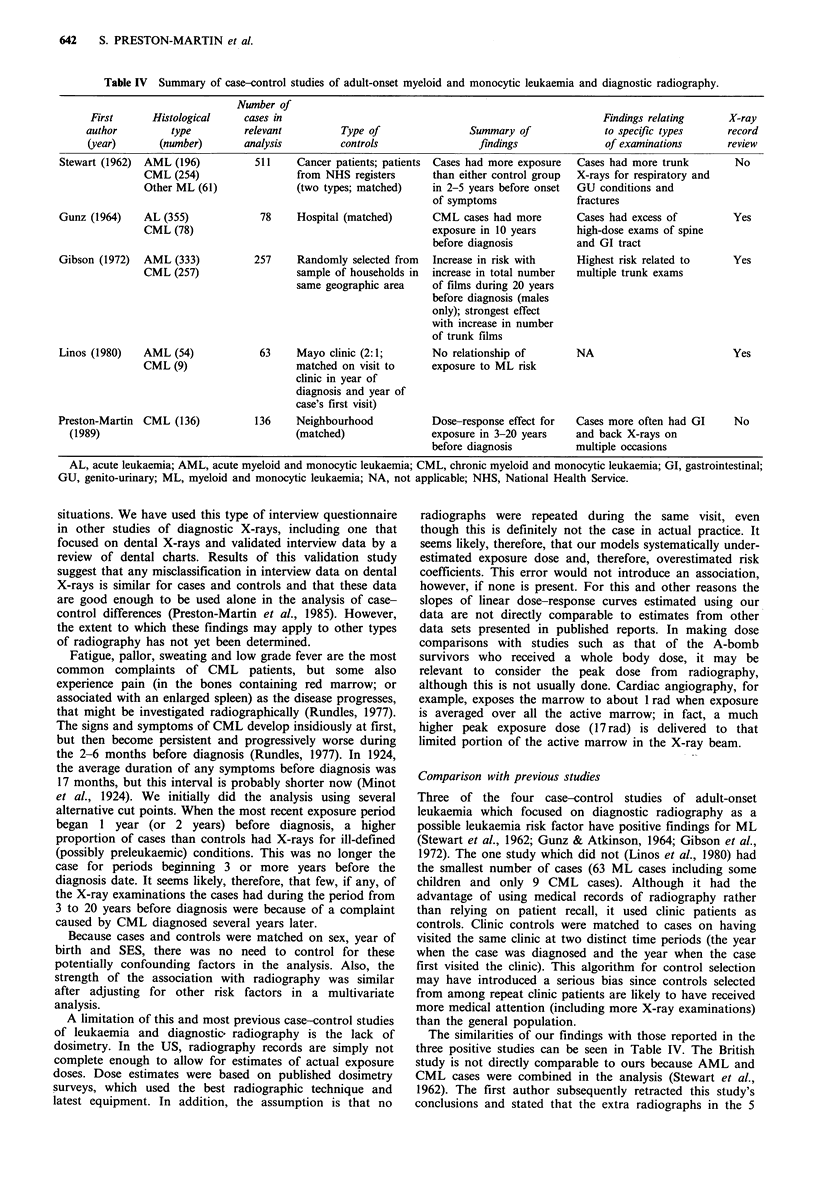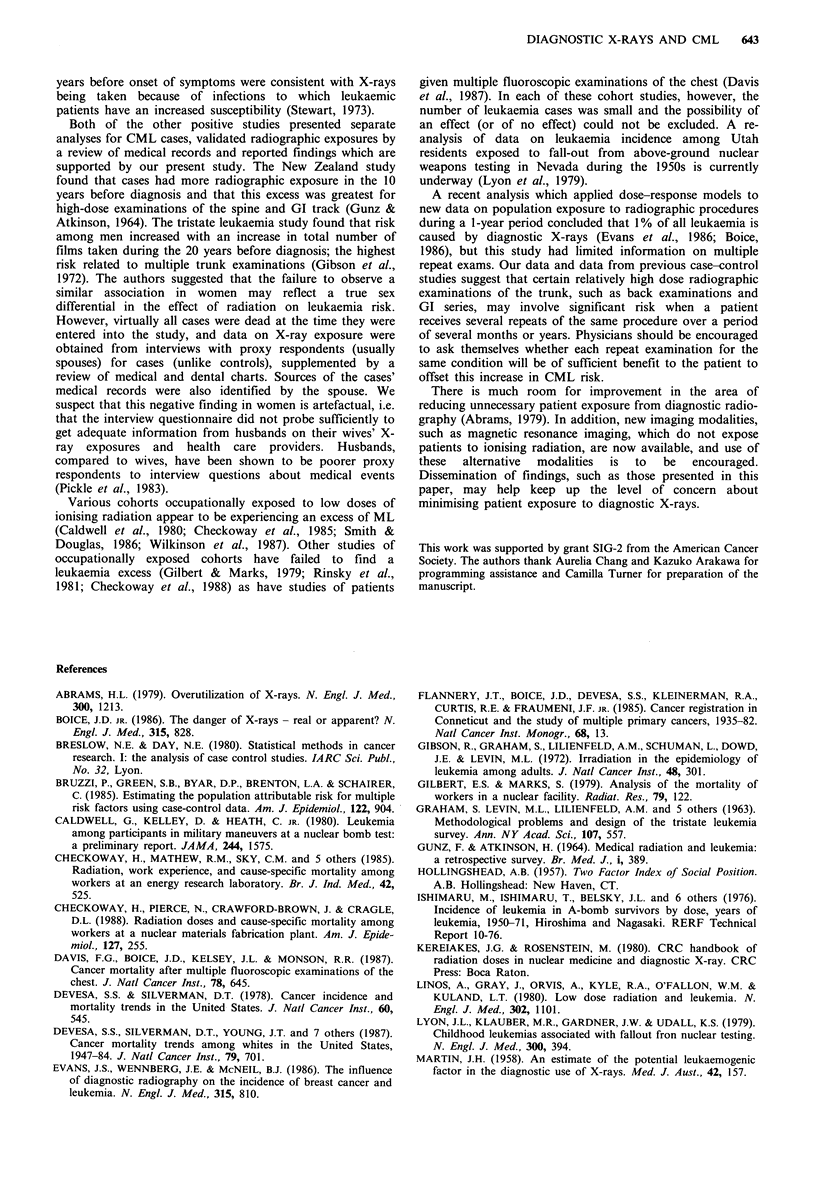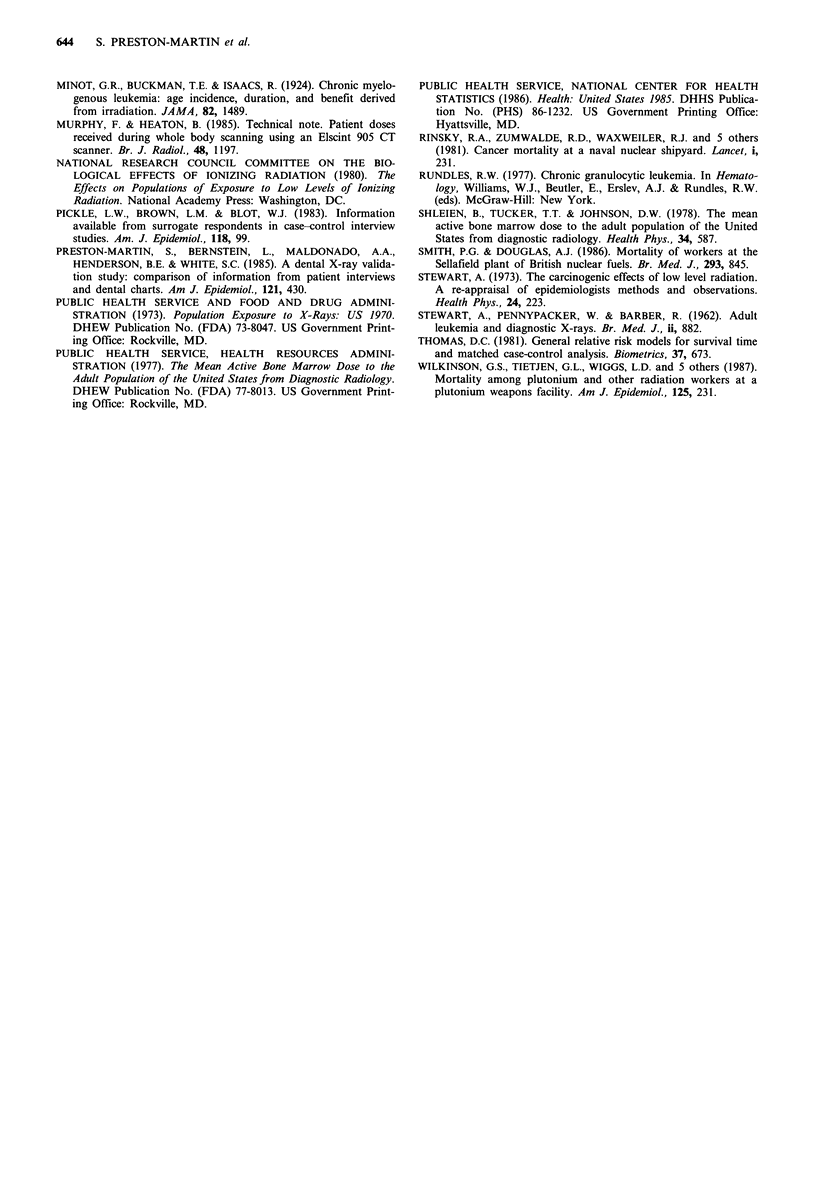# Diagnostic radiography as a risk factor for chronic myeloid and monocytic leukaemia (CML).

**DOI:** 10.1038/bjc.1989.130

**Published:** 1989-04

**Authors:** S. Preston-Martin, D. C. Thomas, M. C. Yu, B. E. Henderson

**Affiliations:** Department of Preventive Medicine, University of Southern California School of Medicine, Los Angeles 90033.

## Abstract

This interview study included 136 Los Angeles County residents aged 20-69 with CML diagnosed from 1979 to 1985 (cases) and 136 neighbourhood controls. During the 3-20 years before diagnosis of the case, more cases than controls had radiographic examinations of the back, gastrointestinal (GI) tract and kidneys, and cases more often had GI and back radiography on multiple occasions (odds ratio (OR) for back X-rays on five or more occasions = 12.0; P less than 0.01). Published estimates were used to assign a minimum dose to the active bone marrow for various radiographic procedures. ORs were estimated for cumulative marrow doses for each of four time periods (3-5 years, 6-10 years, 11-20 years and 3-20 years before the diagnosis of the case). The ORs for exposure to 0.99, 100-999, 1000-1999 and greater than or equal to 2000 mrad in the 3-20 years before diagnosis were 1.0, 1.4, 1.6 and 2.4 (P for highest exposure category and P for trend both less than 0.05). The association was strongest for the period 6-10 years before diagnosis, and the effects of radiation exposure during this period remained significant after consideration of other risk factors in a logistic regression analysis.


Br. J. Cancer (1989), 59, 639–644 © The Macmillan Press Ltd., 1989

### Diagnostic radiography as a risk factor for chronic myeloid and monocytic leukaemia (CML)

S. Preston-Martin, D.C. Thomas, M.C. Yu & B.E. Henderson

*Department of Preventive Medicine, University of Southern California School of Medicine, 2025 Zonal Avenue, Los Angeles, CA 90033, USA.*

**Summary** This interview study included 136 Los Angeles County residents aged 20–69 with CML diagnosed from 1979 to 1985 (cases) and 136 neighbourhood controls. During the 3–20 years before diagnosis of the case, more cases than controls had radiographic examinations of the back, gastrointestinal (GI) tract and kidneys, and cases more often had GI and back radiography on multiple occasions (odds ratio (OR) for back X-rays on five or more occasions = 12.0; $P<0.01$). Published estimates were used to assign a minimum dose to the active bone marrow for various radiographic procedures. ORs were estimated for cumulative marrow doses for each of four time periods (3–5 years, 6–10 years, 11–20 years and 3–20 years before the diagnosis of the case). The ORs for exposure to 0.99, 100–999, 1000–1999 and ≥2000 mrad in the 3–20 years before diagnosis were 1.0, 1.4, 1.6 and 2.4 ($P$ for highest exposure category and $P$ for trend both $<0.05$). The association was strongest for the period 6–10 years before diagnosis, and the effects of radiation exposure during this period remained significant after consideration of other risk factors in a logistic regression analysis.

The aetiology of CML is not well understood, but the occurrence of the Philadelphia chromosome in 90% of cases suggests the importance of agents which cause chromosomal breaks, such as ionising radiation and chemicals. Among A-bomb survivors exposed to relatively low doses (0–99 rads) of gamma-rays, CML was the most common type of leukaemia which developed (Ishimaru *et al.*, 1976). We conducted a population based study of CML in Los Angeles County, California, to investigate the hypothesis that this disease was related to exposure to diagnostic X-rays to the trunk during the 20 years before diagnosis.

Although the rates for all leukaemia combined have not increased substantially in recent decades, rates for myeloid and monocytic leukaemia (ML) have (Devesa & Silverman, 1978; Flannery *et al.*, 1985; Devesa *et al.*, 1987). Our rationale for focusing on this hypothesis related to the secular increase both in the incidence of CML and in population exposure to diagnostic X-rays.

In the United States about half of the population exposure to ionising radiation comes from natural sources (primarily from cosmic rays and terrestrial irradiation) and half from man-made sources. Ninety per cent of population exposure from man-made sources comes from medical X-rays, and diagnostic radiography accounts for most of this (National Research Council, 1980). Although improved radiographic technique has, for some exams (e.g. mammography), led to a reduction in patient exposure, other new techniques expose patients to higher radiation doses. For example, the increased use of grids (lead strips used to absorb secondary radiation and enhance film contrast) increased per film exposure during standard abdominal examination by 30% between 1964 and 1970 (Public Health Service, 1973, 1977). During the same period, the rate of X-ray examinations per 100 people per year rose and the annual per capita mean active bone marrow dose to US adults from radiography increased by 24% (from 83 to 103 mrad) (Public Health Service, 1973). Also of concern is the introduction and rapid increase in use over the past decade of several new radiographic procedures such as CAT scans and cardiac angiography (Public Health Service, 1986).

The exposure to the US population from various X-ray procedures to the trunk (almost 90% of the active bone marrow in adults is located in bones in the trunk (Martin, 1958)) is consistent with the population distribution of CML. Like CML rates, the usage rates of most of the high dose procedures increases steadily with age and with level of education (Public Health Service, 1973), and these rates are higher in men than in women (Public Health Service, 1973, 1986).

Correspondence: S. Preston-Martin.
Received August 1988, and in revised form, November 1988.

### Materials and methods

The patients were Los Angeles County residents, aged 20–69 years, with histologically confirmed CML (ICD-O codes 9863 and 9893) first diagnosed from 1 April 1979 to 30 June 1985. The patients were identified by the University of Southern California Cancer Surveillance Program, the population-based cancer registry for Los Angeles County. As the questionnaire sought detailed information on X-rays received during the 20 years before leukaemia diagnosis, we restricted the study to living patients.

The Cancer Surveillance Program identified 229 eligible cases. Their attending physicians granted permission to contact 206 (90%) of these patients. We were unable to locate 41 patients and 28 patients refused to be interviewed. We obtained completed questionnaires on 137 (83% of the 165 patients contacted about the study or 60% of all eligible cases). Eighty-three patients who would otherwise have been eligible were deceased.

We sought an individually matched neighbourhood control for each of the 137 cases. Each control matched the case in sex, race (black or white) and birth year (within 5 years). Both cases and controls had to be able to be interviewed in English. Matching on neighbourhood of residence resulted in a close match in socioeconomic status (SES) as determined by use of an index which combines information on occupation and level of education (Hollingshead, 1957). To find the controls, we used a procedure that defines a sequence of houses in specified neighbourhood blocks. Our goal was to identify the first matching resident in the sequence and to get his telephone number so that he could be contacted by the interviewer. If no one was at home at the time of visit, we left an explanatory letter and made a follow-up visit after several days. In 102 (74%) instances, the first appropriate person agreed to co-operate. When the first matched control refused to participate, the next in the sequence was sought. In all, 136 matched neighbourhood controls were interviewed.

All interviews were conducted by telephone from June 1982 to February 1986 by four interviewers; both members of a matched pair were interviewed by the same interviewer. Because we explained to each subject how we obtained his or her name, the interviewer was aware of the subject's status. The average length of each interview was 30 min for cases and 24 min for controls. Interview information was obtained up to the date of diagnosis of the case but analyses of X-ray data excluded events which occurred less than 3 years before this date. Six pairs were excluded from these analyses because either the case (3) or the control (3) had a history of radiation treatment to the trunk. One of these six was also the only study subject with a history of cancer chemotherapy.

Our estimates of bone marrow exposure doses from various radiographic procedures were derived primarily from published estimates based on dosimetry models which calculate mean active bone marrow dose to adults from diagnostic radiography and fluoroscopy as practised in the United States in 1970 (Shleien et al., 1978). Other published data were used to estimate bone marrow exposure doses from radioisotope scans and CAT scans (Kereiakes & Rosenstein, 1980; Murphy & Heaton, 1985). We devised a look up table for procedures which assigned the exposure dose indicated for each of several radiographic examinations, including the following: skull, 78 mrad; teeth, 9 mrad; chest, 10 mrad; chest fluoroscopy, 44 mrad; cervical spine, 52 mrad; lumbar spine, 347 mrad ('back', not otherwise specified, was assumed to be lumbar); lumbosacral spine, 450 mrad; stomach and upper GI series with barium swallow, 535 mrad; lower GI series with barium enema, 875 mrad. These estimates are likely to have systematically underestimated patient exposure since, in practice, radiographic technique is commonly less than optimal and it is often necessary to repeat the radiographic procedure on the same visit. We did not attempt to estimate exposure from background sources such as cosmic radiation (which relates primarily to altitude) or from any sources other than those associated with medical and dental care or occupation.

The matched pair design was maintained throughout the analysis. Conditional logistic regression methods were used to determine whether there was a dose-related increase or decrease in risk. In trend tests, factors were always considered as continuous rather than as categorical variables. These multivariate methods were also used to examine the joint effects of several variables. All statistical methods used are described in detail by Breslow & Day (1980). All statistical significance levels ($P$ values) quoted are two-sided and exact 95% confidence intervals (CI) were calculated.

General relative risk models (Thomas, 1981) were used to explore alternatives to the constant relative risk (as a function of age and sex), exponential dose–response model implicit in logistic regression. These included linear dose–response and constant absolute risk models. For example, linear absolute risk model, risk $= r_0$ (age, sex) $+ \beta$ dose, was fitted as relative risk $= 1 + \beta$ dose$/r_0$ (age, sex), where $r_0$ was the age and sex-specific rate of CML for Los Angeles County. Attributable risk calculations were based on the method of Bruzzi et al. (1985).

### Results

The majority of the 130 pairs used in this analysis were male (79 pairs) and most were white (108 pairs). Table I shows the number of cases and controls who ever had each of several common diagnostic radiographic examinations during the period 3–20 years before diagnosis of the case. There was little difference in the number of cases and controls who had at least one routine radiograph of the chest during this period. More cases than controls had X-ray examinations of the spine, GI tract and kidneys. Cases compared to controls more often had GI or back examinations on multiple occasions. Five cases and no controls had GI series (upper and/or lower) on four or more separate occasions. An equal number of cases and controls had gallbladder examinations and more controls than cases had angiography. Ten of the 11 subjects who had angiography had cardiac angiography (1 control had abdominal fluoroscopy). No subject had angiography more than once.

Table II compares cases and controls on the number of separate visits to a medical care provider during which back X-rays were taken. The most striking difference was that 11 cases and only one control had radiographic examinations of the back on five or more occasions (OR = 12.0; $P < 0.01$). The distribution by reason for back examination was similar for cases and controls except that five cases and no controls had arthritis, which was the condition associated with the largest average number of back X-ray visits (7.2 visits). For each of the other reasons for back X-rays (except surgery for which both cases and controls had an average of four back X-ray examinations), the average number of X-ray visits was greater for cases than for controls. The maximum number of radiographic examinations of the back was 22 among cases compared to six among controls, and the respective mean numbers were 5.0 and 2.1. Ten cases and eight controls had jobs which involved some potential for exposure to ionising radiation. The distribution by job type was roughly similar for cases and controls, and the average duration working on these jobs was somewhat longer for controls.

**Table I** Comparison of 130 CML cases and 130 controls by site and number who had each type of diagnostic radiographic examination in the 3–20 years before diagnosis of the case, Los Angeles County, 1979–1985.

| *Site of medical examination* | *Estimated range of dose to active bone marrow per exam (mrad)*[a] | *Total who ever had each type of examination* | |
|---|---|---|---|
| | | *Cases* | *Controls* |
| Routine chest | 10–44 | 115 | 110 |
| All other trunk | | | |
| Back (spine) | 247–749 | 38 | 30 |
| Gallbladder | 129–168 | 5 | 5 |
| GI series | 535–875 | 46 | 38 |
| Kidney | 147–420 | 20 | 12 |
| Angiography | 1133 | 3 | 8 |
| All other procedures | 42–240 | 14 | 10 |
| Other sites | | | |
| Head[b] | 9–78 | 8 | 4 |
| Extremities[c] | 0 | 23 | 14 |

[a]Range of doses are for various radiographic procedures used for that site, e.g. chest X-rays = 10 mrad; chest fluoroscopy = 44 mrad (Shleien et al., 1978; Kereiakes & Rosenstein, 1980; Murphy & Hector, 1985); [b]Does not include dental radiography; [c]Information on radiographs of the limbs was recorded only if patient was X-rayed on more than one occasion. (The dose to the active bone marrow from these examinations was assumed to be zero.)

**Table II** Distribution of CML cases and controls by number of back X-ray visits in the 3–20 years before diagnosis of the case, Los Angeles County, 1979–1985.

| *Total back X-ray visits*[a] | *Cases* | *Controls* |
|---|---|---|
| 1 | 9 | 12 |
| 2 | 8 | 11 |
| 3 | 5 | 3 |
| 4 | 5 | 3 |
| 5 or more | 11 | 1 |
| Total | 38 | 30 |

[a]Total number of visits to a medical care facility for radiographic examinations of the back during time period.

Table III shows dose–response relationships for CML and diagnostic X-rays for each of four time periods before diagnosis of the case (3–5 years, 6–10 years, 11–20 years, 3–20 years). In this analysis, a cumulative dose over the period of interest to the active bone marrow of 0–99 mrad is used as the baseline. (Only three cases and four controls had no X-rays or only a single chest or dental examination in the 3–20 years before diagnosis.) For all periods, an elevation in risk is seen among those whose bone marrow was exposed to 2,000 mrad or more. The strongest association with bone marrow dose was observed for the period 6–10 years before diagnosis of the case; the estimated slopes of the linear relative risk relationships (i.e. the excess relative risks per rad) were 0.76 $rad^{-1}$ ($P<0.05$) for the 6–10 year period and 0.30 $rad^{-1}$ for the 3–20 year period ($P<0.05$). Adjusting for other risk factors (specific genetic syndromes and occupational exposures) increased these slopes only slightly. In a multivariate analysis, the effect of the other two time periods disappeared after adjusting for exposure in the 6–10 year period. We estimate that 17% of the CML cases may be attributable to exposure to diagnostic X-rays to the trunk during the 6–10 years before the date of diagnosis of the case or 23% over the period 3–20 years before.

To assess whether the assumption of a constant relative risk was valid, we examined the slope coefficients separately in each sex and two age groups. The slopes were higher in females and in those under age 50; both of these groups had lower baseline incidence rates, and there was no effect in those over age 50. This finding suggested that an absolute risk model might be more appropriate, and indeed the overall effect was more significant for this model ($\chi^2=7.36$ compared with 6.32 for the relative risk model). The overall unadjusted slope estimates (i.e. the excess risks) using the absolute risk model were 11.5 per $10^6$ person-year-rad for the 6–10 year period and 4.9 per $10^6$ person-year-rad for the 3–20 year period; but risk per rad estimates should be interpreted with caution because cumulative doses may be grossly underestimated. No dosimetry was possible and dose estimates assumed optimal techniques for each exam. In the younger age group, to which the effect was confined, men and women had the same relative risks, but men had a somewhat higher absolute risk; these findings are consistent with those in the A-bomb survivor data.

### Discussion

This study was unable to include a dosimetry component or to validate reported radiography histories by a review of medical charts. We cannot rule out, therefore, the possibility that the difference we observe may be at least partially attributable to biased recall. None the less, these findings are of interest in that they are consistent with those from other studies of CML which were able to validate radiographic exposures. Also, the association we observe in the 3–20 years before diagnosis shows a dose–response effect.

### *Methodological issues*

The potential for bias is a concern for case–control studies of this sort. Although interviewers were aware of the case or control status of each subject, we attempted to minimise interviewer bias through use of a questionnaire with a verbatim script and the prescribed use of a standard set of probes. We also cannot exclude the possibility that recall bias may have occurred. Subjects were told that the study aimed to get information that might tell something about the causes or prevention of certain diseases. Questions were asked about a number of factors including specific drugs, occupations, hobbies and radiography. We tried to minimise recall bias by asking subjects to remember events (e.g. accidents) or conditions (e.g. back pain) which might have necessitated diagnostic X-rays. For all radiography, we asked the patient the reason for the examination, the number of separate occasions on which radiographs were taken and the number of films exposed in each examination. To minimise the effect of any recall bias, the analysis used only 'reason for the exam' and 'total number of X-ray visits' in the calculations of estimated dose. Despite our precautions, the possibility that bias may occur cannot be ruled out because of the lack of blinding of the interviewers and the tendency of cancer patients to focus on the reasons they got cancer. None the less, our findings of an increase in risk related to repeat radiographic examinations of the back or GI tract are of particular interest because they support specific findings from earlier studies of CML which validated interview information by a review of medical records (see below and Table IV).

X-ray examinations are often not particularly memorable events. The tristate leukaemia study, which was conducted in 1960, found that subjects failed to mention about 80% of the X-ray examinations they had in the 10 years before interview (Graham *et al.*, 1963). This finding prompted us to develop a highly structured questionnaire which included series of specific probes to be used in each of several

**Table III** Dose–response relationships for CML and diagnostic X-rays, Los Angeles County, 1979–1985: matched analysis.

| *Minimum cumulative dose to total active bone marrow over indicated period (mrad)* | *Years before diagnosis of case* | | | | | | | |
|---|---|---|---|---|---|---|---|---|
| | *3–5* | | *6–10* | | *11–20* | | *3–20* | |
| Estimated odds ratios and (no. cases/no. controls) | | | | | | | | |
| 0–99 (baseline) | 1.0 | (86/94) | 1.0 | (81/91) | 1.0 | (67/73) | 1.0 | (23/34) |
| 100–999 | 1.1 | (25/25) | 0.9 | (18/26) | 1.1 | (44/44) | 1.4 | (53/55) |
| 1,000–1,999 | 1.7 | (8/5) | 3.1[b] | (19/8) | 0.8 | (8/10) | 1.6 | (22/21) |
| 2,000+ | 2.1 | (11/6) | 2.7[a] | (12/5) | 3.9[b] | (11/3) | 2.4[b] | (32/20) |
| Excess relative risk per rad[c] | 0.29 | | 0.76[b] | | 0.34 | | 0.30[b] | |

[a]$P<0.10$ (two-sided); [b]$P<0.05$; [c]Since cumulative doses are likely to be grossly underestimated, these risk per rad estimates should be interpreted with caution. True excess risks per rad are likely to be considerably lower.

**Table IV** Summary of case–control studies of adult-onset myeloid and monocytic leukaemia and diagnostic radiography.

| *First author (year)* | *Histological type (number)* | *Number of cases in relevant analysis* | *Type of controls* | *Summary of findings* | *Findings relating to specific types of examinations* | *X-ray record review* |
|---|---|---|---|---|---|---|
| Stewart (1962) | AML (196)<br>CML (254)<br>Other ML (61) | 511 | Cancer patients; patients from NHS registers (two types; matched) | Cases had more exposure than either control group in 2–5 years before onset of symptoms | Cases had more trunk X-rays for respiratory and GU conditions and fractures | No |
| Gunz (1964) | AL (355)<br>CML (78) | 78 | Hospital (matched) | CML cases had more exposure in 10 years before diagnosis | Cases had excess of high-dose exams of spine and GI tract | Yes |
| Gibson (1972) | AML (333)<br>CML (257) | 257 | Randomly selected from sample of households in same geographic area | Increase in risk with increase in total number of films during 20 years before diagnosis (males only); strongest effect with increase in number of trunk films | Highest risk related to multiple trunk exams | Yes |
| Linos (1980) | AML (54)<br>CML (9) | 63 | Mayo clinic (2:1; matched on visit to clinic in year of diagnosis and year of case's first visit) | No relationship of exposure to ML risk | NA | Yes |
| Preston-Martin (1989) | CML (136) | 136 | Neighbourhood (matched) | Dose–response effect for exposure in 3–20 years before diagnosis | Cases more often had GI and back X-rays on multiple occasions | No |

AL, acute leukaemia; AML, acute myeloid and monocytic leukaemia; CML, chronic myeloid and monocytic leukaemia; GI, gastrointestinal; GU, genito-urinary; ML, myeloid and monocytic leukaemia; NA, not applicable; NHS, National Health Service.

situations. We have used this type of interview questionnaire in other studies of diagnostic X-rays, including one that focused on dental X-rays and validated interview data by a review of dental charts. Results of this validation study suggest that any misclassification in interview data on dental X-rays is similar for cases and controls and that these data are good enough to be used alone in the analysis of case–control differences (Preston-Martin *et al.*, 1985). However, the extent to which these findings may apply to other types of radiography has not yet been determined.

Fatigue, pallor, sweating and low grade fever are the most common complaints of CML patients, but some also experience pain (in the bones containing red marrow; or associated with an enlarged spleen) as the disease progresses, that might be investigated radiographically (Rundles, 1977). The signs and symptoms of CML develop insidiously at first, but then become persistent and progressively worse during the 2–6 months before diagnosis (Rundles, 1977). In 1924, the average duration of any symptoms before diagnosis was 17 months, but this interval is probably shorter now (Minot *et al.*, 1924). We initially did the analysis using several alternative cut points. When the most recent exposure period began 1 year (or 2 years) before diagnosis, a higher proportion of cases than controls had X-rays for ill-defined (possibly preleukaemic) conditions. This was no longer the case for periods beginning 3 or more years before the diagnosis date. It seems likely, therefore, that few, if any, of the X-ray examinations the cases had during the period from 3 to 20 years before diagnosis were because of a complaint caused by CML diagnosed several years later.

Because cases and controls were matched on sex, year of birth and SES, there was no need to control for these potentially confounding factors in the analysis. Also, the strength of the association with radiography was similar after adjusting for other risk factors in a multivariate analysis.

A limitation of this and most previous case–control studies of leukaemia and diagnostic radiography is the lack of dosimetry. In the US, radiography records are simply not complete enough to allow for estimates of actual exposure doses. Dose estimates were based on published dosimetry surveys, which used the best radiographic technique and latest equipment. In addition, the assumption is that no radiographs were repeated during the same visit, even though this is definitely not the case in actual practice. It seems likely, therefore, that our models systematically underestimated exposure dose and, therefore, overestimated risk coefficients. This error would not introduce an association, however, if none is present. For this and other reasons the slopes of linear dose–response curves estimated using our data are not directly comparable to estimates from other data sets presented in published reports. In making dose comparisons with studies such as that of the A-bomb survivors who received a whole body dose, it may be relevant to consider the peak dose from radiography, although this is not usually done. Cardiac angiography, for example, exposes the marrow to about 1 rad when exposure is averaged over all the active marrow; in fact, a much higher peak exposure dose (17 rad) is delivered to that limited portion of the active marrow in the X-ray beam.

### *Comparison with previous studies*

Three of the four case–control studies of adult-onset leukaemia which focused on diagnostic radiography as a possible leukaemia risk factor have positive findings for ML (Stewart *et al.*, 1962; Gunz & Atkinson, 1964; Gibson *et al.*, 1972). The one study which did not (Linos *et al.*, 1980) had the smallest number of cases (63 ML cases including some children and only 9 CML cases). Although it had the advantage of using medical records of radiography rather than relying on patient recall, it used clinic patients as controls. Clinic controls were matched to cases on having visited the same clinic at two distinct time periods (the year when the case was diagnosed and the year when the case first visited the clinic). This algorithm for control selection may have introduced a serious bias since controls selected from among repeat clinic patients are likely to have received more medical attention (including more X-ray examinations) than the general population.

The similarities of our findings with those reported in the three positive studies can be seen in Table IV. The British study is not directly comparable to ours because AML and CML cases were combined in the analysis (Stewart *et al.*, 1962). The first author subsequently retracted this study's conclusions and stated that the extra radiographs in the 5

years before onset of symptoms were consistent with X-rays being taken because of infections to which leukaemic patients have an increased susceptibility (Stewart, 1973).

Both of the other positive studies presented separate analyses for CML cases, validated radiographic exposures by a review of medical records and reported findings which are supported by our present study. The New Zealand study found that cases had more radiographic exposure in the 10 years before diagnosis and that this excess was greatest for high-dose examinations of the spine and GI track (Gunz & Atkinson, 1964). The tristate leukaemia study found that risk among men increased with an increase in total number of films taken during the 20 years before diagnosis; the highest risk related to multiple trunk examinations (Gibson *et al.*, 1972). The authors suggested that the failure to observe a similar association in women may reflect a true sex differential in the effect of radiation on leukaemia risk. However, virtually all cases were dead at the time they were entered into the study, and data on X-ray exposure were obtained from interviews with proxy respondents (usually spouses) for cases (unlike controls), supplemented by a review of medical and dental charts. Sources of the cases' medical records were also identified by the spouse. We suspect that this negative finding in women is artefactual, i.e. that the interview questionnaire did not probe sufficiently to get adequate information from husbands on their wives' X-ray exposures and health care providers. Husbands, compared to wives, have been shown to be poorer proxy respondents to interview questions about medical events (Pickle *et al.*, 1983).

Various cohorts occupationally exposed to low doses of ionising radiation appear to be experiencing an excess of ML (Caldwell *et al.*, 1980; Checkoway *et al.*, 1985; Smith & Douglas, 1986; Wilkinson *et al.*, 1987). Other studies of occupationally exposed cohorts have failed to find a leukaemia excess (Gilbert & Marks, 1979; Rinsky *et al.*, 1981; Checkoway *et al.*, 1988) as have studies of patients given multiple fluoroscopic examinations of the chest (Davis *et al.*, 1987). In each of these cohort studies, however, the number of leukaemia cases was small and the possibility of an effect (or of no effect) could not be excluded. A re-analysis of data on leukaemia incidence among Utah residents exposed to fall-out from above-ground nuclear weapons testing in Nevada during the 1950s is currently underway (Lyon *et al.*, 1979).

A recent analysis which applied dose–response models to new data on population exposure to radiographic procedures during a 1-year period concluded that 1% of all leukaemia is caused by diagnostic X-rays (Evans *et al.*, 1986; Boice, 1986), but this study had limited information on multiple repeat exams. Our data and data from previous case–control studies suggest that certain relatively high dose radiographic examinations of the trunk, such as back examinations and GI series, may involve significant risk when a patient receives several repeats of the same procedure over a period of several months or years. Physicians should be encouraged to ask themselves whether each repeat examination for the same condition will be of sufficient benefit to the patient to offset this increase in CML risk.

There is much room for improvement in the area of reducing unnecessary patient exposure from diagnostic radiography (Abrams, 1979). In addition, new imaging modalities, such as magnetic resonance imaging, which do not expose patients to ionising radiation, are now available, and use of these alternative modalities is to be encouraged. Dissemination of findings, such as those presented in this paper, may help keep up the level of concern about minimising patient exposure to diagnostic X-rays.

This work was supported by grant SIG-2 from the American Cancer Society. The authors thank Aurelia Chang and Kazuko Arakawa for programming assistance and Camilla Turner for preparation of the manuscript.

**References**

ABRAMS, H.L. (1979). Overutilization of X-rays. *N. Engl. J. Med.*, **300**, 1213.

BOICE, J.D. JR. (1986). The danger of X-rays – real or apparent? *N. Engl. J. Med.*, **315**, 828.

BRESLOW, N.E. & DAY, N.E. (1980). Statistical methods in cancer research. I: the analysis of case control studies. *IARC Sci. Publ., No. 32*, Lyon.

BRUZZI, P., GREEN, S.B., BYAR, D.P., BRENTON, L.A. & SCHAIRER, C. (1985). Estimating the population attributable risk for multiple risk factors using case-control data. *Am. J. Epidemiol.*, **122**, 904.

CALDWELL, G., KELLEY, D. & HEATH, C. JR. (1980). Leukemia among participants in military maneuvers at a nuclear bomb test: a preliminary report. *JAMA*, **244**, 1575.

CHECKOWAY, H., MATHEW, R.M., SKY, C.M. and 5 others (1985). Radiation, work experience, and cause-specific mortality among workers at an energy research laboratory. *Br. J. Ind. Med.*, **42**, 525.

CHECKOWAY, H., PIERCE, N., CRAWFORD-BROWN, J. & CRAGLE, D.L. (1988). Radiation doses and cause-specific mortality among workers at a nuclear materials fabrication plant. *Am. J. Epidemiol.*, **127**, 255.

DAVIS, F.G., BOICE, J.D., KELSEY, J.L. & MONSON, R.R. (1987). Cancer mortality after multiple fluoroscopic examinations of the chest. *J. Natl Cancer Inst.*, **78**, 645.

DEVESA, S.S. & SILVERMAN, D.T. (1978). Cancer incidence and mortality trends in the United States. *J. Natl Cancer Inst.*, **60**, 545.

DEVESA, S.S., SILVERMAN, D.T., YOUNG, J.T. and 7 others (1987). Cancer mortality trends among whites in the United States, 1947–84. *J. Natl Cancer Inst.*, **79**, 701.

EVANS, J.S., WENNBERG, J.E. & MCNEIL, B.J. (1986). The influence of diagnostic radiography on the incidence of breast cancer and leukemia. *N. Engl. J. Med.*, **315**, 810.

FLANNERY, J.T., BOICE, J.D., DEVESA, S.S., KLEINERMAN, R.A., CURTIS, R.E. & FRAUMENI, J.F. JR. (1985). Cancer registration in Conneticut and the study of multiple primary cancers, 1935–82. *Natl Cancer Inst. Monogr.*, **68**, 13.

GIBSON, R., GRAHAM, S., LILIENFELD, A.M., SCHUMAN, L., DOWD, J.E. & LEVIN, M.L. (1972). Irradiation in the epidemiology of leukemia among adults. *J. Natl Cancer Inst.*, **48**, 301.

GILBERT, E.S. & MARKS, S. (1979). Analysis of the mortality of workers in a nuclear facility. *Radiat. Res.*, **79**, 122.

GRAHAM, S. LEVIN, M.L., LILIENFELD, A.M. and 5 others (1963). Methodological problems and design of the tristate leukemia survey. *Ann. NY Acad. Sci.*, **107**, 557.

GUNZ, F. & ATKINSON, H. (1964). Medical radiation and leukemia: a retrospective survey. *Br. Med. J.*, **i**, 389.

HOLLINGSHEAD, A.B. (1957). *Two Factor Index of Social Position.* A.B. Hollingshead: New Haven, CT.

ISHIMARU, M., ISHIMARU, T., BELSKY, J.L. and 6 others (1976). Incidence of leukemia in A-bomb survivors by dose, years of leukemia, 1950–71, Hiroshima and Nagasaki. RERF Technical Report 10-76.

KEREIAKES, J.G. & ROSENSTEIN, M. (1980). CRC handbook of radiation doses in nuclear medicine and diagnostic X-ray. CRC Press: Boca Raton.

LINOS, A., GRAY, J., ORVIS, A., KYLE, R.A., O'FALLON, W.M. & KULAND, L.T. (1980). Low dose radiation and leukemia. *N. Engl. J. Med.*, **302**, 1101.

LYON, J.L., KLAUBER, M.R., GARDNER, J.W. & UDALL, K.S. (1979). Childhood leukemias associated with fallout fron nuclear testing. *N. Engl. J. Med.*, **300**, 394.

MARTIN, J.H. (1958). An estimate of the potential leukaemogenic factor in the diagnostic use of X-rays. *Med. J. Aust.*, **42**, 157.

MINOT, G.R., BUCKMAN, T.E. & ISAACS, R. (1924). Chronic myelogenous leukemia: age incidence, duration, and benefit derived from irradiation. *JAMA*, **82**, 1489.

MURPHY, F. & HEATON, B. (1985). Technical note. Patient doses received during whole body scanning using an Elscint 905 CT scanner. *Br. J. Radiol.*, **48**, 1197.

NATIONAL RESEARCH COUNCIL COMMITTEE ON THE BIOLOGICAL EFFECTS OF IONIZING RADIATION (1980). *The Effects on Populations of Exposure to Low Levels of Ionizing Radiation*. National Academy Press: Washington, DC.

PICKLE, L.W., BROWN, L.M. & BLOT, W.J. (1983). Information available from surrogate respondents in case–control interview studies. *Am. J. Epidemiol.*, **118**, 99.

PRESTON-MARTIN, S., BERNSTEIN, L., MALDONADO, A.A., HENDERSON, B.E. & WHITE, S.C. (1985). A dental X-ray validation study: comparison of information from patient interviews and dental charts. *Am J. Epidemiol.*, **121**, 430.

PUBLIC HEALTH SERVICE AND FOOD AND DRUG ADMINISTRATION (1973). *Population Exposure to X-Rays: US 1970*. DHEW Publication No. (FDA) 73-8047. US Government Printing Office: Rockville, MD.

PUBLIC HEALTH SERVICE, HEALTH RESOURCES ADMINISTRATION (1977). *The Mean Active Bone Marrow Dose to the Adult Population of the United States from Diagnostic Radiology*. DHEW Publication No. (FDA) 77-8013. US Government Printing Office: Rockville, MD.

PUBLIC HEALTH SERVICE, NATIONAL CENTER FOR HEALTH STATISTICS (1986). *Health: United States 1985*. DHHS Publication No. (PHS) 86-1232. US Government Printing Office: Hyattsville, MD.

RINSKY, R.A., ZUMWALDE, R.D., WAXWEILER, R.J. and 5 others (1981). Cancer mortality at a naval nuclear shipyard. *Lancet*, **i**, 231.

RUNDLES, R.W. (1977). Chronic granulocytic leukemia. In *Hematology*, Williams, W.J., Beutler, E., Erslev, A.J. & Rundles, R.W. (eds). McGraw-Hill: New York.

SHLEIEN, B., TUCKER, T.T. & JOHNSON, D.W. (1978). The mean active bone marrow dose to the adult population of the United States from diagnostic radiology. *Health Phys.*, **34**, 587.

SMITH, P.G. & DOUGLAS, A.J. (1986). Mortality of workers at the Sellafield plant of British nuclear fuels. *Br. Med. J.*, **293**, 845.

STEWART, A. (1973). The carcinogenic effects of low level radiation. A re-appraisal of epidemiologists methods and observations. *Health Phys.*, **24**, 223.

STEWART, A., PENNYPACKER, W. & BARBER, R. (1962). Adult leukemia and diagnostic X-rays. *Br. Med. J.*, **ii**, 882.

THOMAS, D.C. (1981). General relative risk models for survival time and matched case-control analysis. *Biometrics*, **37**, 673.

WILKINSON, G.S., TIETJEN, G.L., WIGGS, L.D. and 5 others (1987). Mortality among plutonium and other radiation workers at a plutonium weapons facility. *Am J. Epidemiol.*, **125**, 231.